# L4 Radiculopathy Presenting as Postoperative Femoral Nerve Neuropraxia Following Total Hip Arthroplasty (THA) in the Setting of Hip-Spine Syndrome

**DOI:** 10.7759/cureus.86161

**Published:** 2025-06-16

**Authors:** Caroline Lindsey, Robert E Bilodeau, Christopher Gonzalez, Rahul Ramanathan, Joon Y Lee, Vivek Sharma

**Affiliations:** 1 Department of Orthopedic Surgery, University of Pittsburgh, Pittsburgh, USA; 2 Pittsburgh Orthopedic Research Group (POSR), University of Pittsburgh, Pittsburgh, USA; 3 Orland Bethel Family Musculoskeletal Research Center (BMRC), University of Pittsburgh, Pittsburgh, USA

**Keywords:** hip-spine syndrome, orthopedic complications and orthopedic surgery, orthopedic surgery, total hip arthroplasty complication, total hip arthroplasty (tha)

## Abstract

Hip-spine syndrome (HSS) is the symptomatic degenerative pathology of both the lumbar spine and hip that can lead to diagnostic and therapeutic challenges, as these pathologies can present with overlapping clinical symptoms. There are no definitive best practices for managing HSS, but there is some evidence that the management of hip pathologies prior to spinal pathologies results in better outcomes. This report details a unique patient who had total hip arthroplasty (THA) for osteoarthritis and experienced postoperative femoral nerve-like neuropraxia. Through prudent clinical reasoning and diagnostic evaluation, the patient’s neuropraxia was determined to be of radicular origin rather than an intraoperative injury of the femoral nerve. This challenging diagnosis exemplifies the importance of recognizing concomitant pathology in the hip and spine, as well as performing appropriate diagnostic testing in a patient who underwent THA and presented with postoperative femoral nerve-like neuropraxia.

## Introduction

As the global population continues to age, it is likely that more patients will present with concomitant degenerative changes in the hip and the spine. These pathologies can present with overlapping symptoms such as pain, stiffness, and neurologic deficit, which can obscure the underlying etiology and complicate treatment. Hip-spine syndrome (HSS) is the symptomatic degenerative pathology of both the lumbar spine and hip that can lead to diagnostic and therapeutic challenges [1]. It is estimated that 19%-47% of adults over 60 have spinal stenosis, and 27% of patients 45 years of age and older have radiographic evidence of hip osteoarthritis on imaging [2]. Another study reports that estimates of HSS prevalence are variable and range from 21.2% to 61.5% [3]. Risk factors for osteoarthritis include older age, female gender, obesity, muscle weakness, and joint injury [4]. Additionally, variations in pelvic anatomy such as increased pelvic incidence (PI) can possibly contribute to the development of HSS. Increased PI alters spinopelvic alignment, which leads to compensatory changes in lumbar mechanics and added stress on both the lumbar spine and hip joint, therefore increasing the risk for developing HSS [5]. Literature explores the question of which pathology should be addressed first in cases of HSS, but no consensus has been reached.

## Case presentation

**Figure 1:**
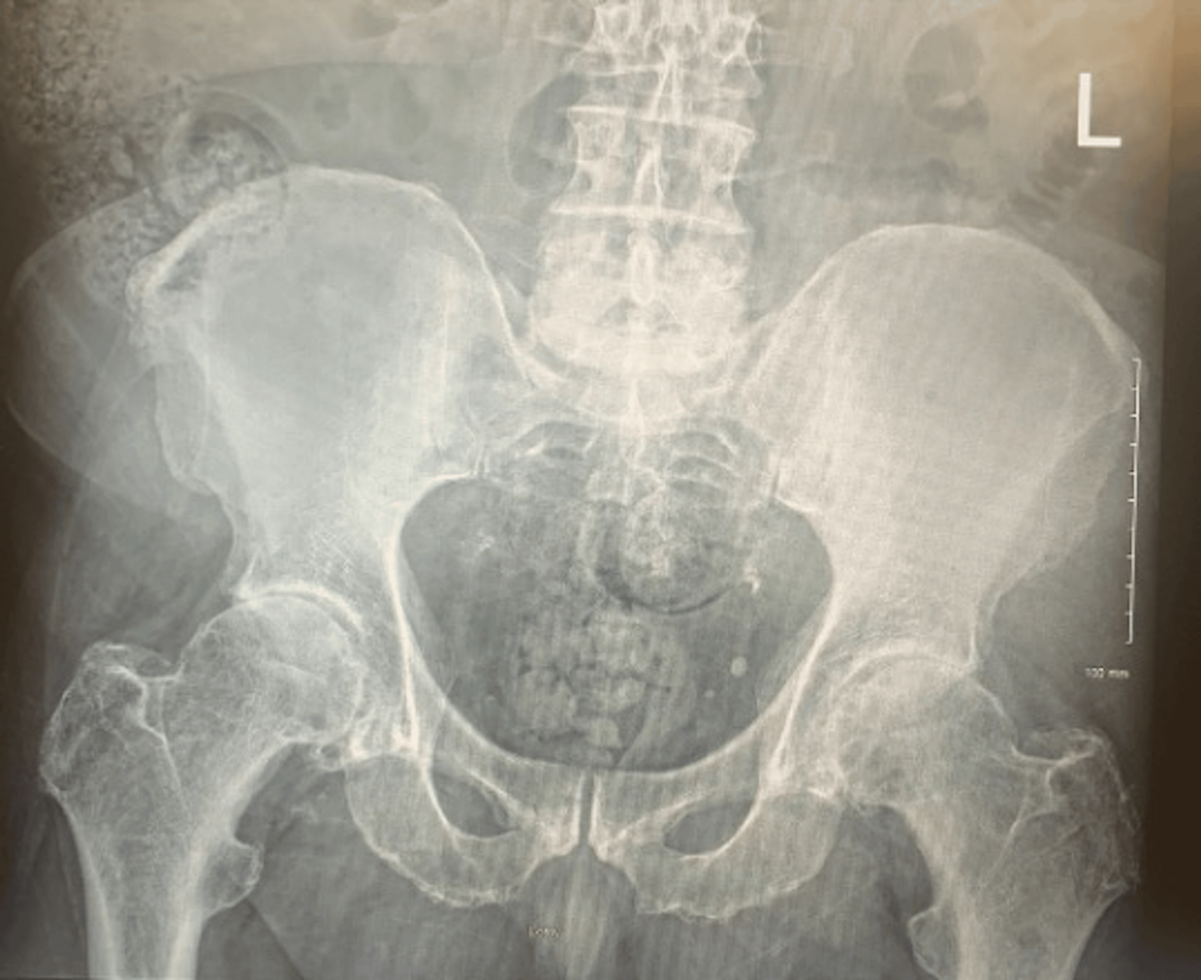
Preoperative anteroposterior radiograph of the pelvis

**Figure 2:**
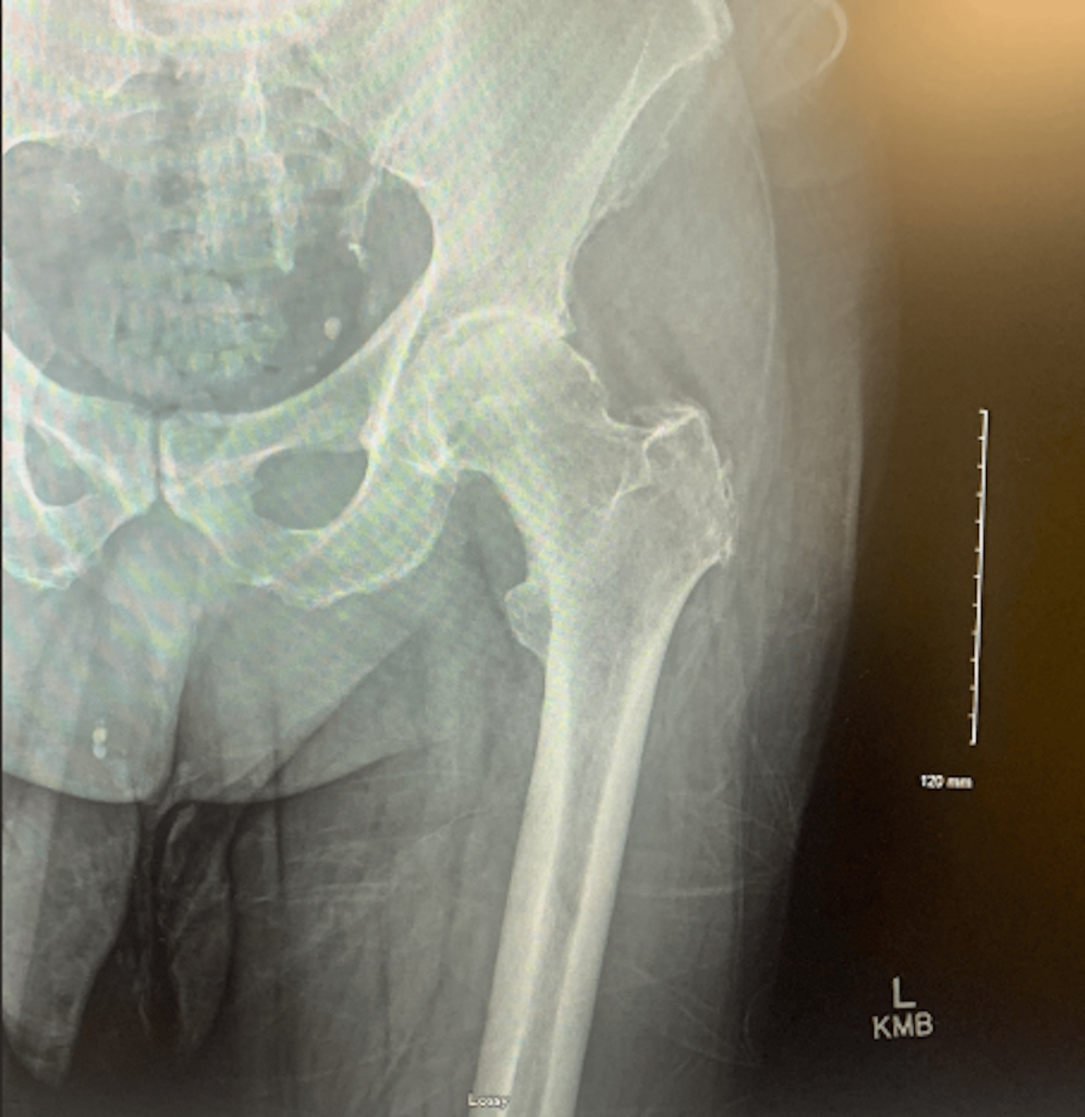
Preoperative anteroposterior radiograph of the left hip

**Figure 3:**
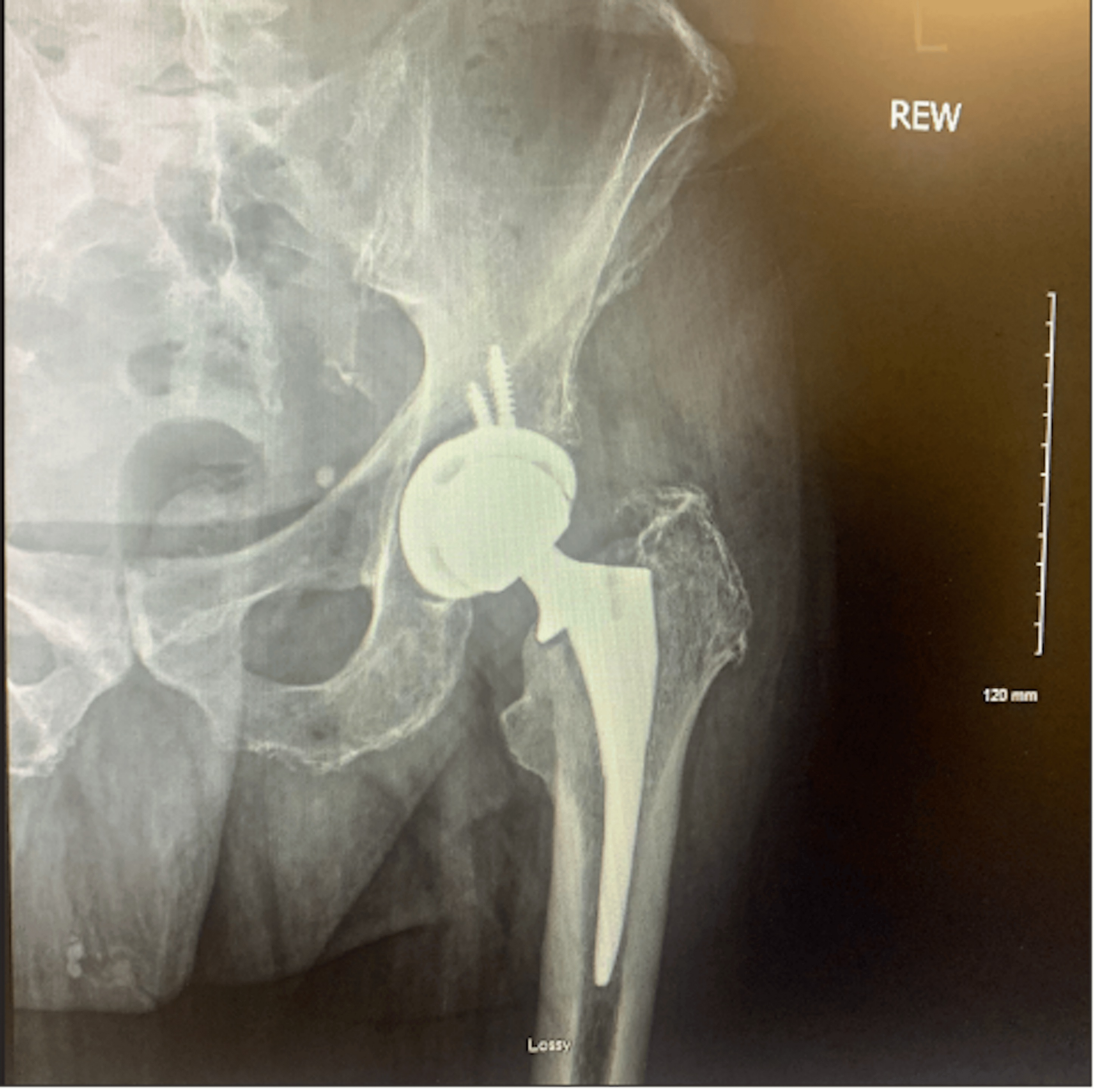
Postoperative anteroposterior radiograph of the left hip

**Figure 4:**
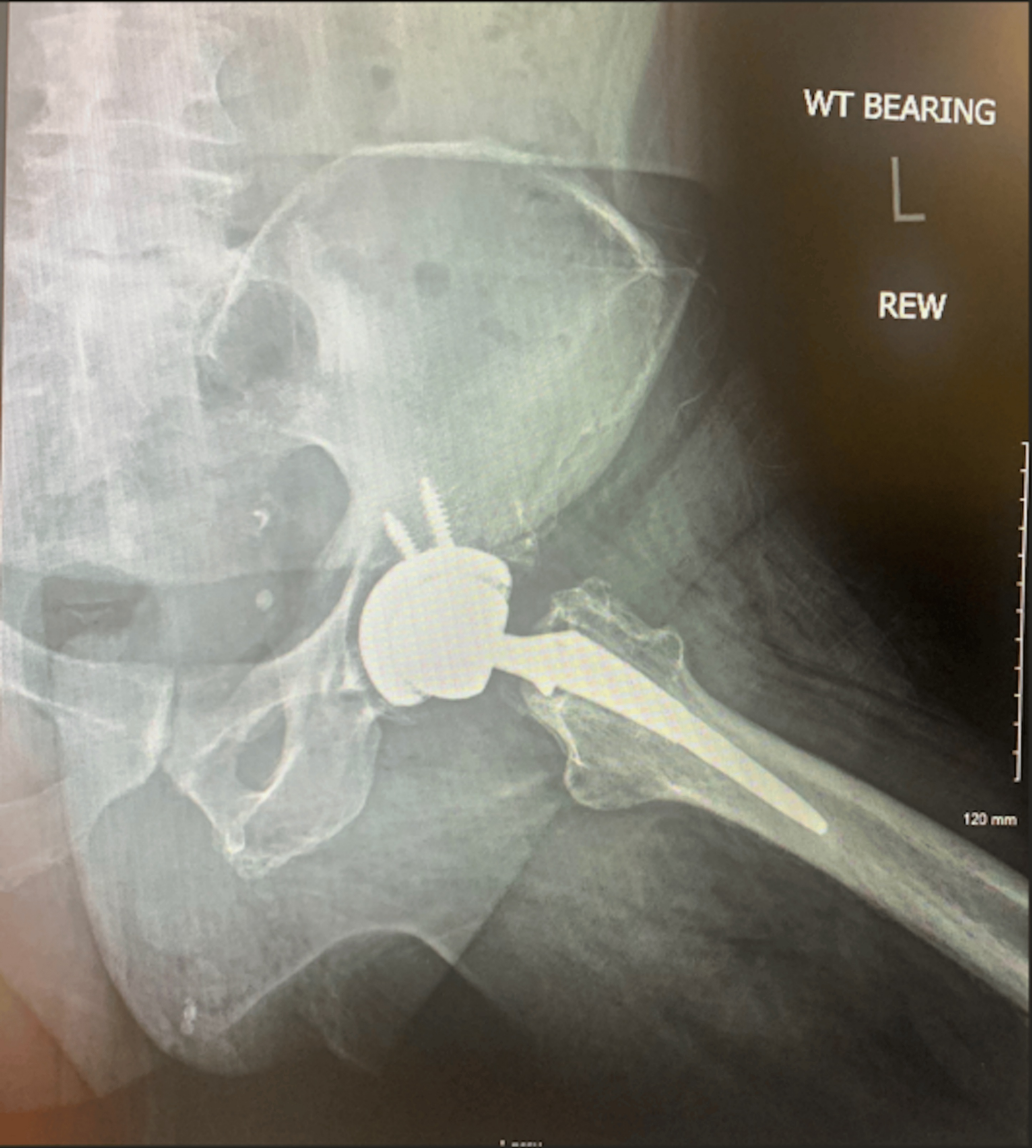
Postoperative Dunn view radiograph of the left hip

A 67-year-old man with hip osteoarthritis presented with severe worsening left hip pain and was unable to ambulate. On examination, he had severe left hip pain with hip rotation and restricted range of motion with 1 cm shortening of the left lower extremity. A detailed spine-focused neurologic examination was deferred due to the suspicion of hip pathology over the spine. X-rays showed severe hip osteoarthritis (Figures 1, 2). The patient elected to proceed with left total hip arthroplasty (THA) using an anterolateral (Hardinge) approach (Figures 3, 4).

At his routine two-week postoperative visit, he was thrilled to be experiencing no hip pain and was ambulating with a cane. He did mention paresthesia and some numbness on the medial aspect of his left shin but reported that the numbness could have been present preoperatively but was masked by pain. On examination, he had weakness of hip flexion and knee extension. These were assumed to be transient muscle weakness due to recent anterior hip surgery. The dorsiflexion and plantarflexion of the ankle were normal. The X-ray showed an uncomplicated THA in satisfactory alignment. The patient was educated on anterior hip precautions and instructed to weight bear as tolerated and ambulate with a cane. A referral to physical therapy (PT) was provided for progressive strengthening and to improve the range of motion in flexion and extension.

At five weeks postoperatively, he was still experiencing persistent numbness in the left leg below the knee anteriorly and posteriorly, which was more obvious to him, and left knee extension weakness, which was worsening. However, he was ambulating well with a cane in his right hand and denied any pain. He demonstrated an upright posture with a level pelvis and no significant leg length discrepancy. Symptoms were not improving despite PT. On examination, left knee extension showed an extensor lag of 10 degrees with grade 4+ strength in knee extension. On sensory examination, the patient had 50% numbness in the left leg below the knee anteriorly and posteriorly to the ankle in the L4 distribution. The neurologic examination of the left ankle and foot was normal. Straight leg test was negative for radicular pain or leg pain. An electromyography (EMG) nerve conduction study was ordered to differentiate femoral nerve neuropraxia from spine etiology. The patient wanted to continue with PT.

**Figure 5:**
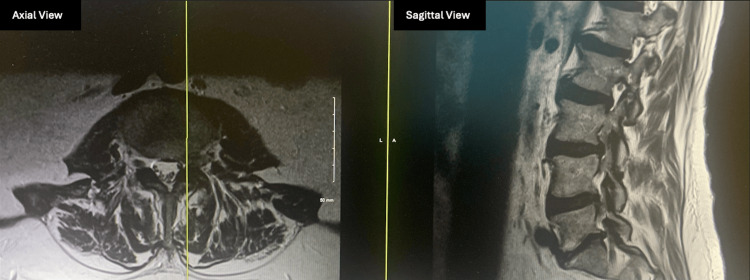
Postoperative MRI axial and sagittal views of the left spine at the area of left-sided stenosis MRI: magnetic resonance imaging

Ten days later, he reported a fall on his back and progressive left leg weakness and paresthesia in the left leg below the knee. On examination, left knee extension was now grade 3, with new ankle dorsiflexor weakness grade 4. He had an absent left knee jerk. He had progressive knee extensor weakness and new-onset ankle and foot weakness and numbness on the anterior medial shin on the left leg, primarily in the L4 dermatome. The straight leg test was negative. Urgent lumbar magnetic resonance imaging (MRI) was ordered to evaluate for compressive neurologic etiology, which showed a left-sided disc extrusion with the compression of L3 and L4 nerve roots with multilevel degenerative disc disease (Figure 5). The EMG results additionally showed no evidence of left lower extremity neuropathy with normal left sural sensory nerve action potential (SNAP) and low-amplitude but normal latency and conduction velocity of left peroneal and tibial compound muscle action potential (CMAP) (Tables 1-4) but rather pointed at L5/S1 radiculopathy. Although the EMG indicated L5/S1 radiculopathy, the findings did not align with the patient’s clinical presentation. His primary weakness was in knee extension, which is more consistent with L3/L4 nerve root involvement. Therefore, based on the clinical examination, rather than EMG, the decision was made to operate at the L3/4 level. Due to progressive neurologic symptoms of weakness and numbness, the patient opted for left-sided L3-L4 laminectomy, microdiscectomy, and foraminotomy for the decompression of L3 and L4 nerve roots. The surgery was performed without complication.

**Table 1:** Motor nerve study: left peroneal nerve

Recording Site: Extensor Digitorum Brevis	Latency (ms)	Duration (ms)	Amplitude (mV)	Area (mVms)	Distance (mm)	Conduction Velocity (m/s)
Ankle	5.1	8.4	0.850	4.0	80	-
Fibular Head	13.0	9.2	1.2	5.8	330	41.7
Popliteal Fossa	15.0	9.1	1.3	6.4	100	50.0

**Table 2:** Motor nerve study: left tibial nerve

Recording Site: Abductor Hallucis	Latency (ms)	Duration (ms)	Amplitude (mV)	Area (mVms)	Distance (mm)	Conduction Velocity (m/s)
Ankle	5.0	3.4	3.1	8.0	80	-
Popliteal Fossa	14.4	9.1	0.350	1.6	400	42.5

**Table 3:** Sensory nerve study: left sural nerve

Recording Site: Ankle	Latency (ms)	Peak Latency (ms)	Amplitude (µV)
Mid-calf	3.3	4.2	7.3

**Table 4:** EMG study EMG: electromyography

Muscle	Insertional Activity	Fibrillations	Positive Spike Waves	Fasciculations	Polyphasic Potentials	Motor Unit Amplitude	Motor Unit Duration	Configuration	Pattern	Recruitment
Left Tibialis Anterior	Normal	Normal	None	None	None	Normal	Normal	Poly	Normal	Normal
Left Peroneus Longus	Normal	Normal	1+	None	None	Normal	Normal	Normal	Normal	Normal
Left Gastrocnemius and Medial Head	Normal	Normal	None	None	None	Normal	Normal	Normal	Normal	-
Left Iliopsoas	Normal	None	None	None	Normal	Normal	Normal	Normal	Normal	Normal
Left Vastus Medialis	Normal	None	None	None	Normal	Normal	Normal	Normal	Normal	-
Left Paraspinals	Normal	None	None	None	Normal	Normal	Normal	Normal	Normal	-

His numbness and weakness improved throughout his postoperative course after spine surgery. Twelve days postoperatively, neurologic testing showed improvement in left knee extension and left foot dorsiflexion from grade 3+ to grade 4. His sensation in the left leg L4 and L5 dermatomes showed improvement to about 50% compared to 25% preoperatively. Left knee jerk testing showed muscle response, which was absent preoperatively. At 6.5 months postoperatively, left knee extension was grade 5 with no extensor lag. He had some patchy numbness on the anteromedial shin with sensation returning to about 75%.

## Discussion

Thorough physical examination and diagnostic evaluation are crucial for identifying and addressing postoperative neurologic deficits following THA. Proper workup requires multifaceted physical, sensory, and advanced imaging tests. Importantly, physicians should not prematurely attribute neurologic deficits after THA to postoperative complications such as femoral nerve injury without a comprehensive assessment of other etiologies [6].

Preoperative differentiation between hip and spine pathology relies on a thorough clinical examination. This can be achieved through the detailed assessment of pain location, provocative maneuvers, and neurologic findings such as strength, reflexes, and sensory changes and their dermatomal patterns [1]. Physical examination in patients presenting with nervous deficit after THA should also include the straight leg raise test (Lasegue test) to assess lumbosacral nerve pathology [7]. Motor and sensory examinations can help differentiate the location of nerve injury, and assessing deep tendon reflexes can point to a specific spinal cord level [8].

In most cases, a thorough clinical examination, including the assessment of pain location, the range of motion, provocative tests, and neurologic signs following dermatomal patterns, is sufficient to determine the primary source of symptoms. However, a neurologic examination does not always provide clear localization of a nerve lesion, especially in cases with overlapping symptoms or subtle deficits, and literature suggests that a negative straight leg raise test does not rule out lumbosacral stenosis [9,10]. When clinical evaluation does not clearly distinguish between hip and spinal pathology, advanced imaging and other diagnostics may be helpful [1]. Clinicians can consider the use of electrodiagnostic testing to assess for nerve conduction injury if the physical examination is inconclusive. EMG allows for the immediate determination of conduction injuries and can assess potential neurologic recovery [11]. Clinicians may also consider advanced imaging such as MRI to assess for spinal pathologies. The use of local injections for the nonoperative management of HSS is not well described in literature. Local injections can be used as a bridge to surgery and can serve as a diagnostic tool for HSS. In clinical practice, the hip is typically injected first; if there is no pain relief, further evaluation is warranted to assess for spinal pathology [1].

Outcomes of patients with HSS are worse than those without coinciding pathology [12]. Recent literature suggests that the management of hip pathologies prior to spinal pathologies reduces lower back pain, reduces the need for lumbar spinal surgery, and increases arthroplasty survival [13-16]. However, this case’s presentation of HSS is unique as neurologic deficits presented only following primary THA, with imaging revealing a left-sided disc herniation at L3-L4 later in his clinical course. Importantly, this case demonstrated the importance of the consideration of HSS as a differential diagnosis in the setting of neurologic deficit following THA.

## Conclusions

This case highlights the diagnostic complexity and clinical implications of HSS. Although the patient initially appeared to have isolated hip osteoarthritis, the subsequent development of progressive neurologic deficits demonstrates the importance of a thorough and ongoing evaluation. This case reinforces the need for clinicians to maintain a high index of suspicion for concomitant spinal disease in cases where symptoms persist or evolve unexpectedly after orthopedic intervention. Ultimately, the early recognition and management of HSS can optimize patient outcomes.
